# Surreptitious M. D’s.

**Published:** 1857-06

**Authors:** 


### EDITORIAL DEPARTMENT.

### SURREPTITIOUS M. D's.

It has been said that the most perfect liberty is found in obedience to law. To the truth of the proposition we have nothing to oppose, although we think the American people are practically opposed to the principle, choosing rather to adopt as a cardinal article of their political faith the motto, "the best government is that which governs least."

In doing this they, unconsciously perhaps, by their anxiety to do away with individual exclusive privileges, run into the extreme of failing to protect and secure that which is of vastly greater importance—the greatest good of the greatest number. In fact, the *tendency* is to the extreme in this direction, and paradoxical as it might appear, this extreme is practically what we attempt to avoid, enlargement of individual rights and lessened protection to society.

But a few years since it was thought to be one of the paramount necessities of well-regulated governments, to clothe the professions of law and medicine with certain immunities and rights not possessed by other members of society. This was esteemed salutary and right, not because it acted as a protection to the individuals of these professions, but because rulers and law makers esteemed the protection of property and lives of the governed of sufficient importance to demand it. Members of these professions obtained the privileges of such regulation, not by virtue of any hereditary or natural right, but they were compelled to pay a high price in time, study and money in order to their acquisition.

But all these are things of the past. "Young America" scouts such things as obsolete—as emanations from the "old fogyism" of its *dis*respected sire. He pronounces all such distinctions between professions and trades as wholly pernicious and opposed to the "inalienable rights of man." He consider that, as "competition is the life of trade," and as there is no tariff, protective or otherwise, for the farmer or merchant, so should there be none for the physician. Put the one precisely on the footing of the other. Let each be held accountable for his contracts, whether to adjust a fractured thigh or patch a boot, and all will be as well. In fact, he looks upon all legal distinctions as odious, simply because he does not appreciate that they are made for the purpose of securing the welfare of society rather than to subserve individual interests.

And what is the result? Read!

"H. C. ——, M. D., Geo. —— M. D."

"Drs. ——— & ———" having associated themselves in the practice of Medicine, Surgery and Obstetrics, in all its various branches, would say to the citizens of —— and surrounding country that they will be ready at all hours to wait upon the unfortunate.—We flatter ouselves that we understand the diseases of the western country—we have had considerable experiences of the diseases peculiar to the western country, and therefore we can make this suggestion—all we want is a trial.

N. B.—They may be found at Dr. ——'s Office, when not professionally engaged.

REFERENCES.

Prof. Hughes, Keokuk, Iowa.
" McDowell, St. Louis.
" Muzzy, Cincinnati.
" Dudley, Lexington, Ky.
" Baker, Cincinnati, O.

————, 1856."

Or this

MEDICAL NOTICE.

"Dr. E. M. —— would respectfully announce to the citizens of ——— and vicinity, that he has located in the above place for the purpose of practising Medicine, Surgery, Obstetrics and Dentistry, in all their various branches. Feeling confident from six years' assiduous study, *and filling the requirements of the Medical Colleges, both East and West,* that he can render satisfaction to all who may favor him with their patronage. He would further state that he has been practising five years and thinks he understands the diseases incident to this country. He will also keep on hand a good assortment of fresh Medicines, *consisting of the leading articles, Castor Oil, Turpentine, &c.* He will keep surgical instruments for sale of the best quality.

REFERENCES.

Prof. Hughes, Iowa University.
" McDowell, St. Louis.
" Baker, Cincinnati, Ohio.

———, July —, 1856."

Now "the greatest freedom" permits, of course, the free use of the alphabet, whether as initials or as indices of titles, to any individual who may feel disposed to so employ them. True others may be foolish enough to suppose the letters thus appended to the name are used by virtue of a legitimate title to some degree which they designate, but of course he is not responsible for such groundless inferences.

And then as to the references to eminent men, most of whom are ignorant of the existence, even, of the individual referring to them—has not one an indisputable right to refer to whom he will? But, seriously, can impudence go further? And is it not time we should have learned how far less culpable it were to abolish all law for the protection of property, than thus give free license to those to whom the lives their fellows are of so much less value than the dollar they obtain as a fee?

But if law-makers refuse to act in this regard, there is yet one step which the Profession and Colleges may, and indeed ought to take in this matter, and that is not only to discountenance such acts, but to expose the actors.

It has become so common a thing for students, upon the completion of their first course of lectures, to go out and establish themselves in practice, and with many of them to resort to such dishonest means as the above, for the purpose of deceiving the public, that it is time for the Medical Colleges to speak out in the matter.

For one we are glad that the Medical Department of the Iowa University has declared its intention to publish as deceivers all of its students who shall hereafter dare to do this thing. The people demand this much of us, that where there is no law to protect them from imposition, the Profession should afford them the protection of its influence against it. Let it be given them, say we. But we cannot close this article without referring to the great laxity of legal see p. 159 (contrast to)

enactments for the security of life and health in our own country, which is apparent in recent occurrences in France. We learn from the journals that the French courts have lately imposed both fine and imprisonment upon venders and makers of Patent Nostrums, on the ground of their being, to all intents and purposes, swindlers. Their advertisements state falsehoods, since they misstate the character of the medicine and also the effects produced thereby, and money thus obtained is procured upon false pretenses. It were well if the example were to be followed by some better governments.

