# Errata &c. in Our Last Number

**Published:** 1857-08

**Authors:** 


*Errata &c., in our First Number.*—We were annoyed exceedingly upon the appearance of our first number to find that the types had made us talk much more erroneously and nonsensically than is our wont even. But the fault was not our own, nor yet that of the printer. We should have seen the proof sheets, but from absence during a part of the time could not see them all, and the compositor was excusable for most of the consequent errors. In an editorial article upon "Surreptitious M. D's.," in the last line of page 72, was an omission between the words *great* and *laxity* of the words *contrast to*, whereby the whole sentence was rendered meaningless. Several other errors we have noticed, but hope hereafter to find no such annoyances to contend with.

The "Times Book and Job Printing Office," which publishes our journal, we believe to be one of the best in the Northwest, and we know there is no office anywhere which can boast of more competent or courteous and gentlemanly employees than friend O. S. Eastman, who attends to the typography and press work of our Journal. The typographical appearance of our present number we will let speak for the office.

